# Heat Transfer Analysis of Nanostructured Material Flow over an Exponentially Stretching Surface: A Comparative Study

**DOI:** 10.3390/nano12071204

**Published:** 2022-04-04

**Authors:** Mubashar Arshad, Azad Hussain, Ali Hassan, Ilyas Khan, Mohamed Badran, Sadok Mehrez, Ashraf Elfasakhany, Thabet Abdeljawad, Ahmed M. Galal

**Affiliations:** 1 Department of Mathematics, University of Gujrat, Gujrat 50700, Pakistan; azad.hussain@uog.edu.pk (A.H.); muhammadali0544@gmail.com (A.H.); 2 Department of Mathematics, College of Science Al-Zulfi, Majmaah University, Al-Majmaah 11952, Saudi Arabia; i.said@mu.edu.sa; 3 Department of Mechanical Engineering, Faculty of Engineering & Technology, Future University in Egypt, New Cairo 11845, Egypt; mohamed.badran@fue.edu.eg; 4 Department of Mechanical Engineering, College of Engineering at Al Kharj, Prince Sattam bin Abdulaziz University, Al-Kharj 16273, Saudi Arabia; sadok_mehrez@yahoo.fr; 5 Department of Mechanical Engineering, University of Tunis El Manar, ENIT, BP 37, Le Belvédère, Tunis 1002, Tunisia; 6 Mechanical Engineering Department, College of Engineering, Taif University, 11099, Taif 21944, Saudi Arabia; a.taha@tu.edu.sa; 7 Department of Mathematics and Sciences, Prince Sultan University, Riyadh 11586, Saudi Arabia; 8 Department of Medical Research, China Medical University, Taichung 40402, Taiwan; 9 Mechanical Engineering Department, College of Engineering, Prince Sattam Bin Abdulaziz University, Wadi Addawaser 11991, Saudi Arabia; ahm.mohamed@psau.edu.sa; 10 Production Engineering and Mechanical Design Department, Faculty of Engineering, Mansoura University, Mansoura 35516, Egypt

**Keywords:** nanofluid, heat transfer, three-dimensional flow, exponential surface, silver, zinc, copper, nanoparticles

## Abstract

The objective of the present research is to obtain enhanced heat and reduce skin friction rates. Different nanofluids are employed over an exponentially stretching surface to analyze the heat transfer coefficients. The mathematical model for the problem has been derived with the help of the Rivilin–Erickson tensor and an appropriate boundary layer approximation theory. The current problem has been tackled with the help of the boundary value problem algorithm in Matlab. The convergence criterion, or tolerance for this particular problem, is set at 10^−6^. The outcomes are obtained to demonstrate the characteristics of different parameters, such as the temperature exponent, volume fraction, and stretching ratio parameter graphically. Silver-water nanofluid proved to have a high-temperature transfer rate when compared with zinc-water and copper-water nanofluid. Moreover, the outcomes of the study are validated by providing a comparison with already published work. The results of this study were found to be in complete agreement with those of Magyari and Keller and also with Lui for heat transfer. The novelty of this work is the comparative inspection of enhanced heat transfer rates and reduced drag and lift coefficients, particularly for three nanofluids, namely, zinc-water, copper-water, and silver-water, over an exponentially stretching. In general, this study suggests more frequent exploitation of all the examined nanofluids, especially Ag-water nanofluid. Moreover, specifically under the obtained outcomes in this research, the examined nanofluid, Ag-water, has great potential to be used in flat plate solar collectors. Ag-water can also be tested in natural convective flat plate solar collector systems under real solar effects.

## 1. Introduction

Problems of flow over an exponentially stretching surface have gained the admirable attention of researchers due to their wide range of applications in many fields, such as engineering and geophysics. Major applications linked to these types of fluid flow is in fluid dynamics, which is the study of natural arising flow, commonly on the earth’s crust. Geophysical fluid flow problems cover a wide range of flow applications. Micro and macro turbulence in the Upper Ocean, as well as convection in clouds, are typically regarded as foci in hydrological, oceanographic, and meteorological studies, respectively [1]. Sakadias [2] developed the boundary layer idea for continually stretching surfaces. Crane [3] made a valuable contribution to Sikaidi’s concept. For steady boundary layer flow, he proposed the notion of both linear and exponentially stretching surfaces and obtained an identical equivalent solution in closed analytical form. Researchers have examined the characteristics of fluid flow with different externally applied effects on three-dimensional movement over a linear and exponentially stretching surface. Hussain et al. [4] have investigated the combined impact of non-linear radiation and magnetic fields on three-dimensional flow across the linear stretching surface. They discovered the Au-water and Ag-water nanofluid heat transfer rates. Nandeppanavar et al. [5] performed a theoretical analysis of Casson nanofluid flow over an exponentially stretching surface for thermal transmission. Arshad et al. [6] discussed the MHD nanofluid movement across an exponentially stretching surface using the Brownian effect. Hussain et al. [7] have explored three-dimensional rotating nanofluid over a stretching surface with a magnetic field for heat transportation.

Numerous industries require high-efficiency heat transfer coefficients as a basic requirement. By mixing metallic or non-metallic particles that are nanometers in size, typically around 100 nm, in a conventional fluid, such as water, oil, etc., the formed mixture is known as nanofluid. Common fluids, such as water, ethylene glycol, kerosene, and oils have much lower thermal conductivity when compared with nanofluid. This distribution of nanoparticles in the host base fluid enhances the thermal conductivity of the nanofluid, resulting in $K_{nf}\geq K_{f}$. This transition of base fluid to a nanofluid not only improves the thermal conductivity of the fluid but as a result, enhances rates of heat transfer significantly. Choi and Eastman [8] introduced the concept of enhancement of thermal conductivity; they coined the term “nanofluid”. The achieved outcome showed that with high thermal conductivity, the pumping power of heat exchangers reduces dramatically. For the last couple of years, researchers have been interested in nanofluid heat convection. 

Sheikhoeslami et al. [9] have investigated the effect of magnetic field on nanofluids for heat transfer using the GMDH-type neural network technique. Khan et al. [10] have explored 3D flow of nanofluids over an exponentially stretching sheet. Mustafa et al. [11] have discussed the impact of the magnetic field on the Casson fluid over a stretching sheet. Rashidi et al. [12] have examined the homotopy solution of nanofluid over the non-linear stretching permeable sheet. Abu-Hamda et al. [13] have introduced a significant note on solar energy employing Powell Eyring nanofluid considering thermal jump conditions along with the implementation of the Cattaneo-Christov heat flux model. Aouinet et al. [14] have investigated the hydrodynamic flow of nanofluids and turbulent boundary layers over a plate. Waqas et al. [15] have explored the Marangoni bio-conventional flow of Reiner–Philippoff nanofluid with a melting phenomenon and a non-uniform heat source and sink in the presence of the swimming microorganism. Alazwari et al. [16] have discussed entropy generation using the classical Keller Box technique for first grade viscoelastic nanofluid over a stretching sheet.

Khan and Pop [17] described the phenomena of nanofluid over a stretched surface for a steady boundary layer. Researchers have investigated for both Newtonian and non-Newtonian nanofluid models. Akbar et al. [18] discussed numerically the stagnation point flow of tangent hyperbolic fluid over the stretchable surface. Hussain et al. [19] described the slip effect on rotating nanofluid over stretching surface. Anuar et al. [20] presented a stability analysis of a stretching surface with a suction effect. Hussain et al. [21] studied the Jeffry fluid flow above an exponentially enlarging surface in two dimensions. The two-dimensional stagnation point flow above an exponentially expanding surface with a temperature fascination effect was reported by Malvandi et al. [22]. Bachok et al. [23] have described the heat and mass transfer analysis of three-dimensional stagnation point nanofluid flow. Recently, Krishna et al. [24] discussed the Blasius and Sakiadis flow with variable properties using thermal convection phenomena. Veera et al. [25] described the heat transmission for copper and alumina-based nanofluid over permeable enlarging surfaces. Gangadhar et al. [26] investigated the transverse flow of hybrid nanofluid using activation energy. Prasad et al. [27] analyzed the variable transport characteristics of Casson fluid with a slip flow phenomenon. Variable diffusivity and conductivity of Williamson fluid flow over an exponentially stretching surface is discussed by Salahuddin et al. [28]. Rehman et al. [29] performed a comparative analysis of second-grade fluid for heat and mass transmission. Kumar et al. [30] discussed MHD slip movement and chemical reaction with joule heating impact over an exponentially stretching surface. Ahmad et al. [31] explained the 3D movement of Maxwell fluid above an exponentially stretching surface with a chemical reaction. Many researchers [32,33,34,35,36,37,38,39] have investigated the characteristics like heat transfer coefficients and reduced skin frictions by using different geometries and effects. Eid [40] used a dual-phase nanofluid model to deliberate the chemical reaction effects on MHD boundary layer movement above an exponentially stretched sheet. Using finite element simulation, Ali et al. [41] demonstrated the importance of Lorentz force on water-based nanofluid and silver nanoparticles. Gopal et al. [42] discussed the MHD nanofluid movement with oh-mic effects, chemical reactions, and viscous dissipation.

The main objective of the present research is to investigate the three-dimensional flow of nanofluid over an exponentially stretching surface. Water is utilized as a base fluid while three different nanoparticles, namely zinc, copper, and silver, are considered for this investigation. The governing equations of momentum and energy are achieved with the help of Naiver–Stokes and boundary layer approximations. The highly non-linear partial differential equations are then transformed into a system of ordinary differential equations using a suitable similarity transformation. The boundary value problem technique is used to tackle the reduced system of ordinary differential equations in Matlab. The criterion for convergence for the present problem is set at 10^−6^. When present outcomes are compared with already published literature, the achieved results of the enhanced heat transfer coefficient were found to be in complete agreement with previous literature data. The impact of different study parameters is computed on different profiles, such as velocities and temperatures. Then the achieved outcomes are presented graphically and in a tabulated data set.

## 2. Statement of Problem

**Figure 1:**
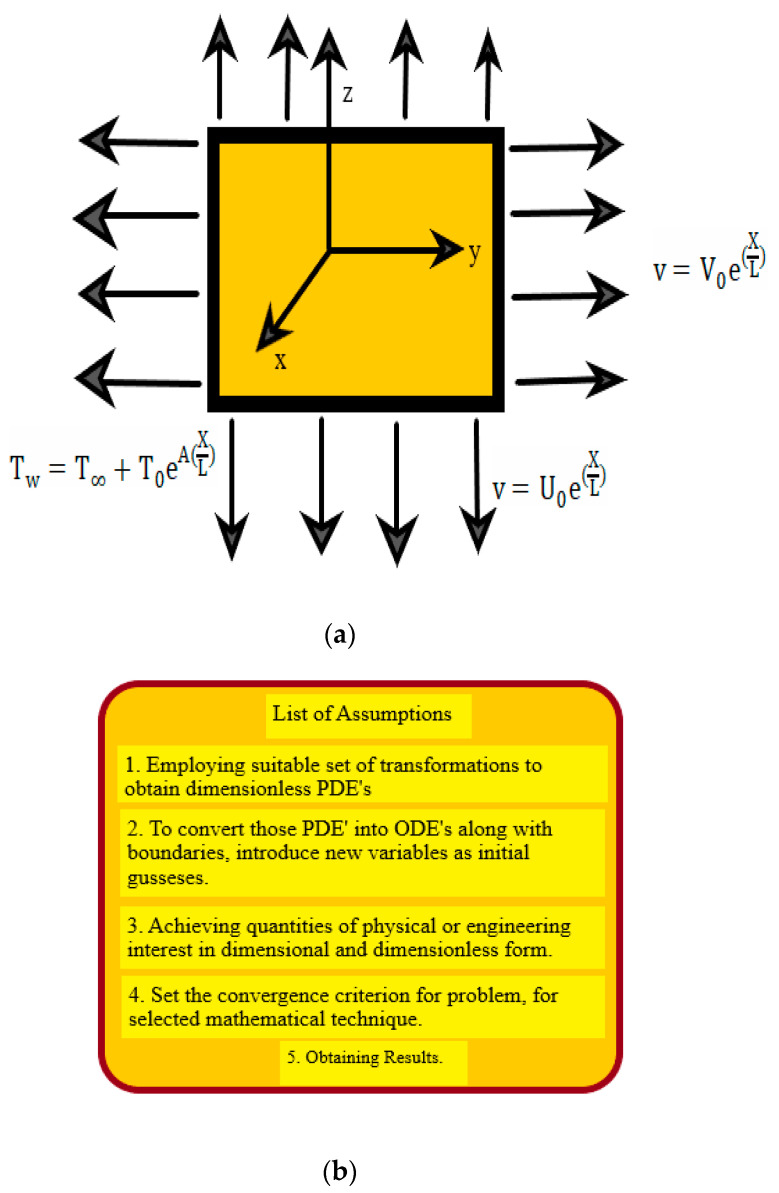

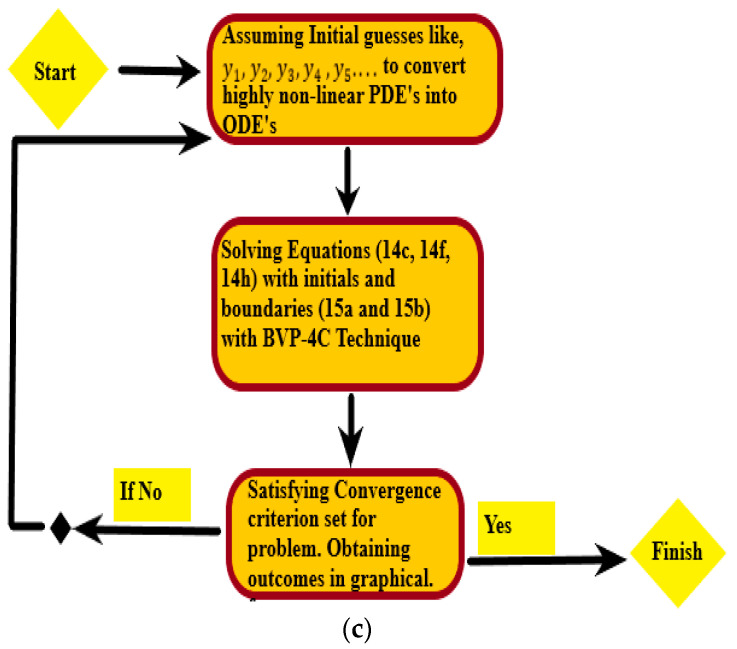
(**a**): The geometry of the problem. (**b**): Assumptions sketch to solve flow governing equations. (**c**): Flow chart of problem illustrating complete cycle.

Assume a three-dimensional laminar, boundary-layer movement over an exponentially extending sheet, with $U_{w}$ and $V_{w}$ being the velocity components in two Cartesian plane directions (x,y), whereas the fluid along the z−axis is considered to be at rest as shown in Figure 1a. The assumptions drawn to solve the problem equation are given below in Figure 1b. $T_{w}$ is the temperature at the wall and $T_{\infty }$ is the temperature of the free stream. The boundary layer approximation theory approximates the flow governing equations. The continuity, momentum, and energy equations (neglecting body forces) of constant physical characteristics are as follows:

(1) $$\frac{\partial u}{\partial x}+\frac{\partial v}{\partial y}+\frac{\partial w}{\partial z}=0,$$

(2) $$u\;\frac{\partial u}{\partial x}+v\frac{\partial u}{\partial y}+w\;\frac{\partial u}{\partial z}=\frac{\mu_{nf}}{\rho_{nf}}\frac{\partial^{2}u}{\partial z^{2}},$$

(3) $$u\;\frac{\partial v}{\partial x}+v\;\frac{\partial v}{\partial y}+w\;\frac{\partial v}{\partial z}=\frac{\mu_{nf}}{\rho_{nf}}\frac{\partial^{2}v}{\partial z^{2}},$$

(4) $$u\;\frac{\partial T}{\partial x}+v\;\frac{\partial T}{\partial y}+w\;\frac{\partial T}{\partial z}=\alpha_{nf}\frac{\partial^{2}T}{\partial z^{2}}.$$

Subjected to the succeeding borderline conditions

(5) $$u=U_{w},\;\;v=V_{w},\;\;w=0,\;\;T=T_{w}\;\;\;\;\;\;\;at\;z=0$$

(6) $$u\to 0,\;\;v\to 0,\;\;T\to T_{\infty },\;\;\;\;\;\;\;\;\;\;\;\;\;\;\;\;\;\;\;\;\;\;\;\;\;\;\;\;\;\;\;as\;z\to \infty$$

In the above expressions u,  v,  and w are velocity components along x,  y, and z−*axes*, respectively. T is the fluid’s temperature, $\rho_{nf}$ is the density of nanofluid, $\alpha_{nf}$ is the thermal diffusivity of nano $\;\mu_{nf}$ is the viscosity of nanofluid.

All of these are linked to nanoparticle’s particle volume fraction ϕ [40].

(7) $$\mu_{nf}=\frac{\mu_{f}}{{\left(1-\phi \right)}^{\frac{5}{2}}}$$

(8) $$\rho_{nf}=\rho_{f}\left(1-\phi \right)+\phi \rho_{s}$$

(9) $${\left(\rho C_{\;p}\right)}_{nf}={\left(\rho C_{\;p}\right)}_{f}\;\left(1-\phi \right)+\phi {\left(\rho C_{\;p}\right)}_{s}$$

(10) $$\frac{k_{f}}{k_{nf}}=\frac{k_{s}\;+\;2k_{f}+\;2\phi \left(k_{f}-k_{s}\right)}{k_{s}\;+\;2k_{f}-\;2\phi \;\left(k_{f}\;-\;k_{s}\right)}$$

(11) $$\upsilon_{nf}=\frac{\mu_{nf}}{\rho_{nf}}$$

(12) $$\alpha_{nf}=\frac{k_{nf}}{{\left(\rho C_{\;p}\right)}_{\;nf}}.$$

Here, $k_{nf}\;$ is the thermal conductivity of nanofluid, ${\left(\rho C_{\;p}\right)}_{nf}$ denotes heat capacity at constant pressure and ϕ is the volumetric concentration of nanoparticles. L is the orientation length, and A is the temperature exponent, where $U_{0},\;V_{0}$, and $T_{0}$ are constants. We define the similarity transformation as:

(13) $$u=U_{0}e^{\left(\frac{X}{L}\right)}f^{\prime }\left(\eta \right),v=V_{0}e^{\left(\frac{X}{L}\right)}g^{\prime }\left(\eta \right),$$

(14) $$w={\left(\frac{vU_{0}}{2L}\right)}^{\frac{1}{2}}e^{\left(\frac{X}{L}\right)}\left\{f\left(\eta \right)+\eta f^{\prime }\left(\eta \right)+g\left(\eta \right)+\eta g^{\prime }\left(\eta \right)\right\}$$

(15) $$T_{w}=T_{\infty }+T_{o}e^{\frac{A\left(X\right)}{L}}\;h\left(\eta \right),\;\;\eta ={\left(\frac{U_{0}}{2yL}\right)}^{\frac{1}{2}}e^{\left(\frac{X}{L}\right)}z$$

By using the above-mentioned similarity transformations Equations (13)–(15), the continuity Equation (1) of the problem will be uniformly satisfied, equations of momentum and energy Equations (2)–(4) incorporating Equations (7)–(12) will take the following form:

(16) $$f^{\prime \prime \prime }=\left[2\left(f^{\prime }+g^{\prime }\right)f^{\prime }-\left(f+g\right)f^{\prime \prime }\right]\left[{\left(1-\phi \right)}^{\frac{5}{2}}\left\{1-\phi +\left(\frac{\rho_{s}}{\rho_{f}}\right)\phi \right\}\right],$$

(17) $$g^{\prime \prime \prime }=\left[2\left(f^{\prime }+g^{\prime }\right)g^{\prime }-\left(f+g\right)g^{\prime \prime }\right]\left[{\left(1-\phi \right)}^{\frac{5}{2}}\left\{1-\phi +\left(\frac{\rho_{s}}{\rho_{f}}\right)\phi \right\}\right],$$

(18) $$h^{\prime \prime }=Pr\left[A\left(f^{\prime }+g^{\prime }\right)h-\left(f+g\right)h^{\prime }\right]\left[{\left(1-\phi \right)}^{5/2}\left\{1-\phi +\phi \;\left(\frac{{\left(\rho C_{\;p}\right)}_{s}}{{\left(\rho C_{\;p}\right)}_{f}}\right)\right\}\right],$$

Subjected to the following transformed boundary conditions Equations (5) and (6), which becomes;

(19) $$f\left(0\right)=0,\;\;f^{\prime }\left(0\right)=1,\;\;g\left(0\right)=0,\;\;g^{\prime }\left(0\right)=\gamma ,\;\;h\left(0\right)=0\;\mathrm{at}\;\eta =\infty$$

(20) $$f^{\prime }\to 0,\;\;g^{\prime }\to 0,\;\;h\to 0,\;\;as\;\eta \to \infty$$

The differentials are w.r.t $\eta ,\;Pr=\frac{{\left(\mu C_{\rho }\right)}_{f}}{k_{f}}$ is Prandtl number and $\gamma =\frac{V_{0}}{U_{0}}$ is the stretching ratio parameter.

## 3. Quantities of Physical Interest 

Skin frictions $C_{fx},C_{fy}$, and local Nusselt number $\;Nu_{x}$ are parameters of physical importance. In dimensional form these parameters are defined as:

(21) $$C_{fx}=\frac{\tau_{wx}}{\rho_{f}U_{0}{}^{2}/2},\;\;C_{fy}=\frac{\tau_{wy}}{\rho_{f}U_{0}{}^{2}/2},\;\;Nu_{x}=\frac{xq_{w}}{k_{f}\left(T_{w}-T_{\infty }\right)}.$$

The wall shear stress is supplied by $\tau_{wx}=\tau_{zx}{|}_{z=0}$ and $\tau_{wy}=\tau_{zy}{|}_{z=0}$ and $q_{w}$ is wall temperature flux given by

(22) $$\tau_{wx}=\mu_{nf}\frac{\partial u}{\partial z}{|}_{z=0}\;,\;\tau_{wy}=\mu_{nf}\frac{\partial v}{\partial z}{|}_{z=0}\;,\;q_{w}=-k_{nf}\frac{\partial T}{\partial z}{|}_{z=0}.$$

The dynamic viscosity and thermal conductivity of nanofluid are represented by $\mu_{nf}$ and $\;k_{nf}$, respectively. The following results for skin frictions in the x- and y-direction and Nusselt number are obtained by using the similarity transformation Equations (13)–(15) in the dimensionless form:

(23) $$\frac{{\left(Re_{x}\right)}^{\frac{1}{2}}C_{fx}}{2e^{\frac{3\left(X\right)}{2L}}}=\frac{1}{{\left(1-\phi \right)}^{\frac{5}{2}}}\;f^{\prime \prime }\left(0\right)$$

(24) $$\frac{{\left(Re_{x}\right)}^{\frac{1}{2}}C_{fy}}{2e^{\frac{3\left(X\right)}{2L}}}=\frac{1}{{\left(1-\phi \right)}^{\frac{5}{2}}}\;g^{\prime \prime }\left(0\right),$$

(25) $$\frac{{\left(Re_{x}\right)}^{-\frac{1}{2}}\;Nu_{x}}{e^{\frac{\left(X\right)}{2L}}}\frac{L}{x}=-\frac{k_{nf}}{k_{f}}\;h^{\prime }\left(0\right).$$

Here Reynolds number is $Re=U_{0}L/2v$ and X=x+y and Y=x−y.

## 4. Numerical Procedure

A coupled system of the ordinary differential Equations (16)–(18) with boundary conditions (19, 20) is utilized in Matlab via the boundary value problem technique. The complete procedure is adopted as follow by defining a new set of variables:

(26) $$f=y\left(1\right),\;f^{\prime }=y\left(2\right),f^{\prime \prime }=y\left(3\right),f^{\prime \prime \prime }=y^{\prime }\left(3\right)$$

(27) $$g=y\left(4\right),\;g^{\prime }=y\left(5\right),g^{\prime \prime }=y\left(6\right),g^{\prime \prime \prime }=y^{\prime }\left(6\right)$$

(28) $$h=y\left(7\right),\;h^{\prime }=y\left(8\right),h^{\prime \prime }=y^{\prime }\left(8\right)$$

The above-given system of differential equations is reduced in first-order ODEs as follows:

(29) $$y^{\prime }\left(1\right)=y\left(2\right)$$

(30) $$y^{\prime }\left(2\right)=y\left(3\right)$$

(31) $$y^{\prime }\left(3\right)={\left(1-\phi \right)}^{2.5}\left(1-\phi +\phi \;\frac{\rho_{s}}{\rho_{f}}\right)\;*\left(\left(\left(2*y\left(2\right)+y\left(5\right)\right)*y\left(2\right)\right)-\left(\left(y\left(1\right)+y\left(4\right)\right)*y\left(3\right)\right)\right)$$

(32) $$y^{\prime }\left(4\right)=y\left(5\right)$$

(33) $$y^{\prime }\left(5\right)=y\left(6\right)$$

(34) $$y^{\prime }\left(6\right)={\left(1-\phi \right)}^{2.5}\left(1-\phi +\phi \;\frac{\rho_{s}}{\rho_{f}}\right)\;*\left(\left(\left(2*y\left(2\right)+y\left(5\right)\right)*y\left(5\right)\right)-\left(\left(y\left(1\right)+y\left(4\right)\right)*y\left(6\right)\right)\right)$$

(35) $$y^{\prime }\left(7\right)=y\left(8\right)$$

(36) $$y^{\prime }\left(8\right)=(pr*\left(A*\left(y\left(2\right)+y\left(5\right)\right)*y\left(7\right)\right)-\left(pr*\left(y\left(1\right)+y\left(4\right)*y\left(8\right)\right)\right)\;*{\left(1-\phi \right)}^{2.5}\left(1-\phi +\phi \;\frac{\rho Cp_{s}}{\rho Cp_{f}}\right)$$

The corresponding boundary conditions are:

(37) y(1)=0,  y(2)=1,  y(4)=0,  y(5)=γ,  y(7)=1    at y=0

(38) y(2)=0,  y(5)=0,  y(7)=0                as y→∞

Table 1 and Table 2 compare the dimensionless temperature transfer rates h′(0) in the absence of nanoparticles (ϕ=0) with the literature [43,44]. Both outcomes are considered to be satisfactory. As a consequence, the authors believe that the current findings are correct as compared to already published literature. Table 1 shows that for any value of Pr and temperature exponent A with γ=0, there is outstanding agreement. Table 2 determines that changing the stretching ratio parameter γ results in good agreement. The standard solution convergence rate is set to be 10^−6^. The flow chart of the whole procedure is shown in Figure 1c. At first, initial guesses were considered, and then Equations (31), (34) and (36) are solved with boundary conditions Equations (37) and (38) using the boundary value problem technique in MATLAB. 

## 5. Results and Discussion

In this section, the outcomes of distinct study parameters, namely, stretching ratio, temperature exponent, and volume fraction of nanoparticles, are elaborated. The results obtained for skin coefficients in the x- and *y-directions* along with the heat transfer coefficient termed as Nusselt number are presented in tabulated data sets. The thermophysical characteristics of the base fluid and solid nanoparticles are shown in Table 3.

**Figure 2:**
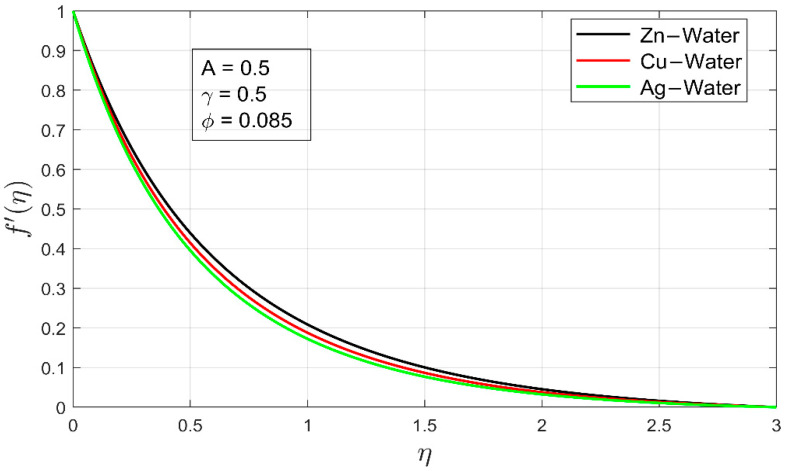
Comparison of velocity profiles $f^{\prime }\;\left(\eta \right)$ for different nanofluid.

**Figure 3:**
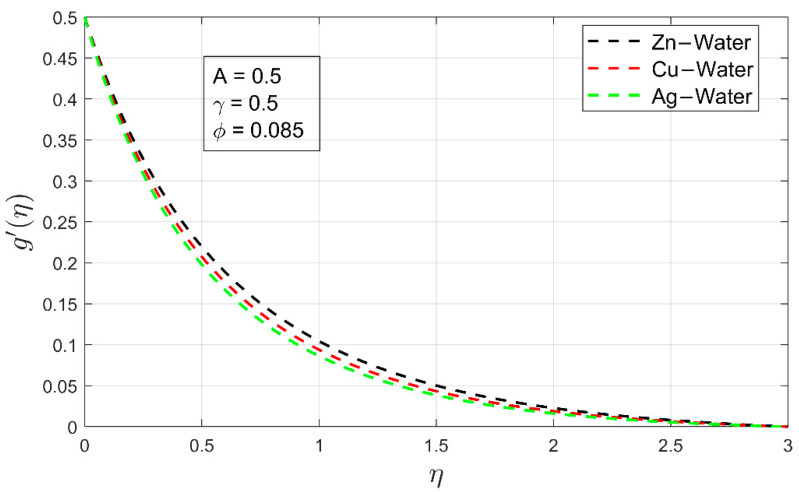
Comparison of velocity profiles $g^{\prime }\;\left(\eta \right)$ for different nanofluid.

Figure 2 and Figure 3 show the velocity profiles for Zn-water,  Cu-water and Ag-water nanofluid, respectively. In both figures, it can be observed that the velocity of Zn-water nanofluid is higher as compared to other nanofluids. It is due to the lower density of Zn nanoparticles. The latter Zn-water can flow easily as compared to the Cu-water and Ag-water. Although overall, both velocity profiles in their respective directions are linearly decreasing, resulting in a decline of the associated momentum boundary layers of freely moving nanofluids by keeping the stretching ratio and thermal exponent at constant rates. This fact also illustrates that the momentum boundary layer of a freely moving fluid is directly affected by the volume fraction of the nanoparticle. To keep the outcome more accurate, a minimal volume fraction of all nanoparticles are utilized. 

**Figure 4:**
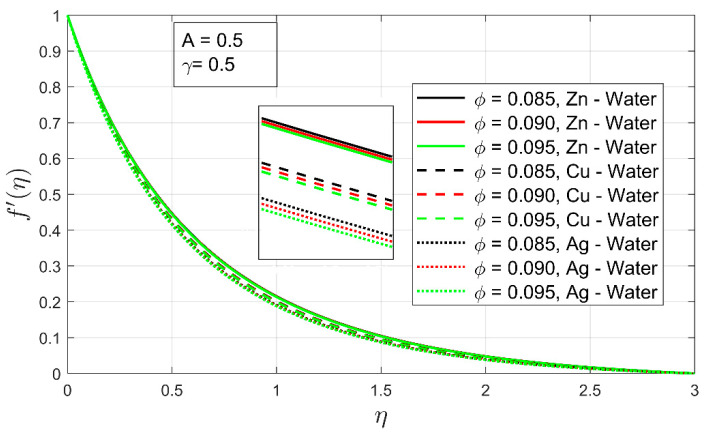
The influence of volume fraction ϕ on velocity profile $f^{\prime }\left(\eta \right)$.

**Figure 5:**
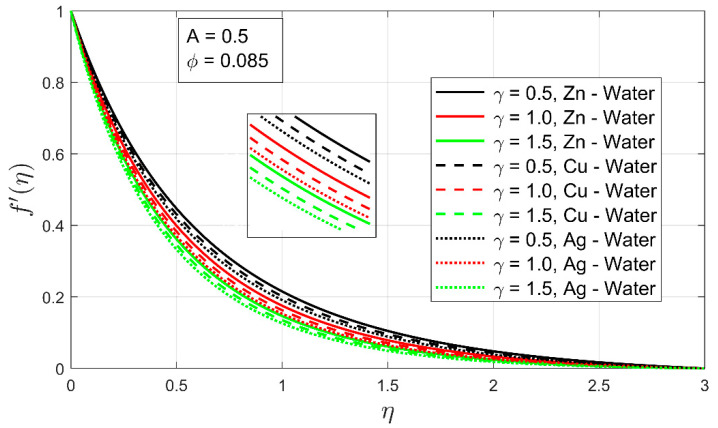
The influence of stretching ratio γ on velocity profile $f^{\prime }\left(\eta \right)$.

Figure 4 and Figure 5 demonstrate the impacts of the volumetric concentration ϕ and the stretching ratio parameter γ on the velocity profile $f^{\prime }\left(\eta \right)$, respectively. The velocity profile $\;f^{\prime }\left(\eta \right)$ is found to decrease when the particle volume ϕ percentage is increased. This phenomenon occurs as the volume fraction ϕ of the nanoparticles increases, the fluid becomes thicker, and an opposing force arises that leads toward deceleration (Figure 4). The overall velocity  Zn-water is higher following the same argument as in Figure 2. It can easily be observed that the momentum boundary layer of nanofluids has contracted. Minimal momentum layer contraction can be seen in the case of Ag-water. The increment in volume fraction of the nanoparticles contributes to the decrease of the momentum boundary layer, eventually decreasing the velocity profile. Similarly, the velocity profile $f^{\prime }\left(\eta \right)$ decreases as the stretching ratio parameter γ increases because of an inverse relationship between $\gamma \;\mathrm{and}\;\;f^{\prime }\left(\eta \right)$. In the case of Figure 5, when γ upsurges, the stretching rate along the y-axis rises as compared to the x-axis. So, the fluid’s motion reduces along the x-axis, which shows deceleration in the velocity profile $f^{\prime }\left(\eta \right)$. Furthermore, it is observed that with an increment in stretching ratio, the momentum layer for all nanofluid cases has expanded, whereas the opposite behavior was observed with an increment in volume fraction. This opposite behavior indicates that the stretching ratio greatly influences the motion of freely moving fluid. Moreover, the expansion observed in the case of Zn-water, this case was higher as compared to other nanofluids examined in the study. 

**Figure 6:**
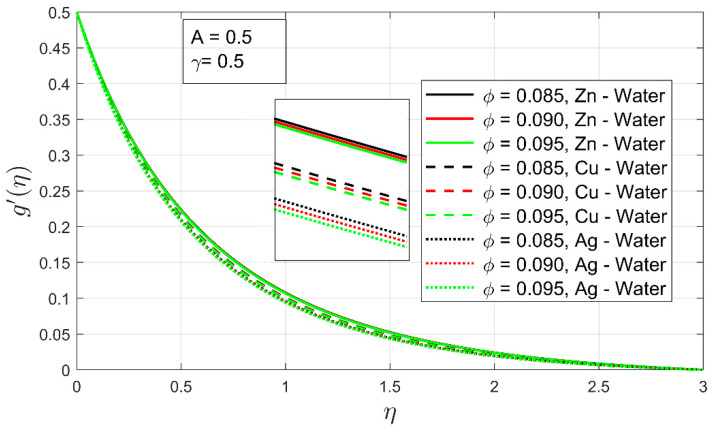
The influence of volume concentration ϕ on the velocity profile $g^{\prime }\left(\eta \right)$.

**Figure 7:**
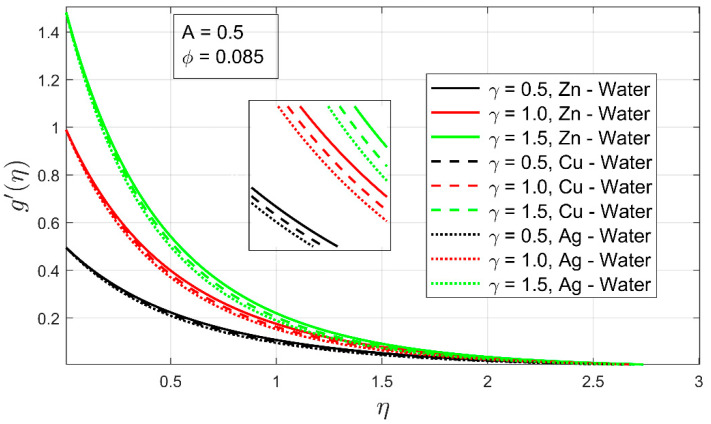
The influence of stretching ratio γ on the velocity profile $g^{\prime }\left(\eta \right)$.

In Figure 6 and Figure 7, the influence of volume fraction ϕ and stretching ratio constraint γ on the velocity profile $g^{\prime }\left(\eta \right)$ is shown. The increment in the volume fraction of solid nanoparticles has decreased the secondary velocity profile. As a result, the associated momentum layer has also contracted. This phenomenon has been observed in all cases of examined nanofluids. It can be easily noted that if a higher concentration of volume fraction is employed, the thickness of the boundary layer upsurges, which instantly decreases the secondary velocity profile and associated boundary layer (See Figure 6). The velocity profile $g^{\prime }\left(\eta \right)$ is found to be higher in the case of Zn-water and for Cu-water nanofluid. Because zinc particles have a lower density as compared to copper and gold particles, so, they can flow easily when mixed in water. Therefore, the Zn-water velocity profile falls slowly as compared to other nanofluids (see Figure 6).

Figure 7 shows that as the stretching ratio parameter γ rises, the velocity profile $g^{\prime }\left(\eta \right)$ rises. This behavior is noticed due to the boundary condition $g^{\prime }\left(0\right)=\gamma$. It is interesting to notice that the thickness of the boundary layer has increased sharply as the bi-linear stretching is increased. The lower volume fraction and constant thermal exponent are major contributors to the enhancement of the associated boundary layer. The secondary velocity profile has increased as stretching is rising.

**Figure 8:**
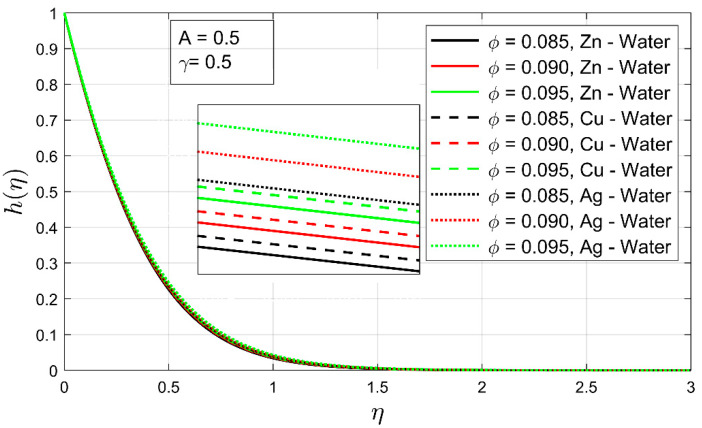
The temperature profile h(η) as a function of volume concentration ϕ.

**Figure 9:**
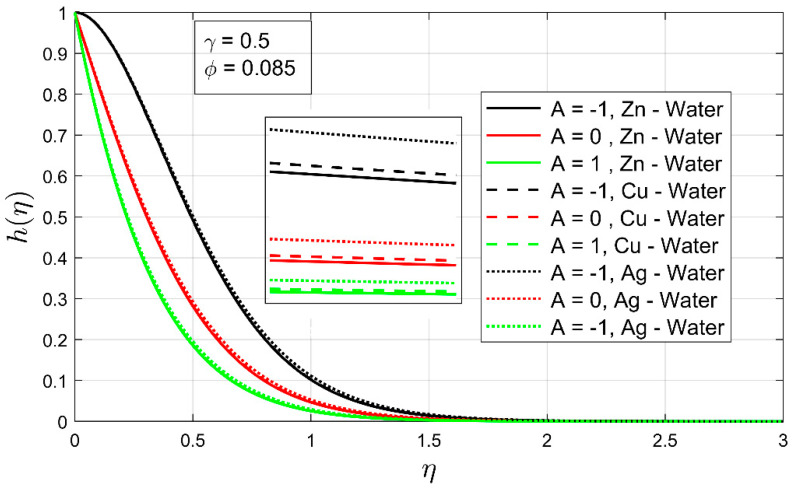
The temperature profile h(η) as a function of temperature exponent A.

**Figure 10:**
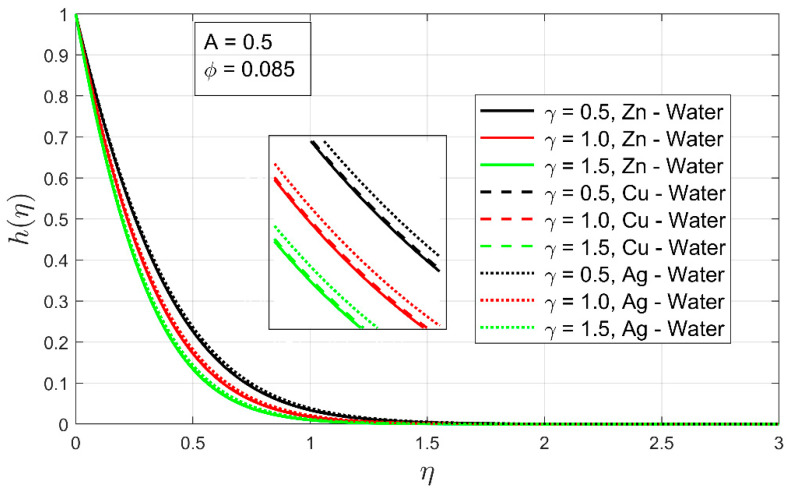
The temperature profile h(η) as a function of stretching ratio parameter γ.

The impact of volume fraction, thermal exponent, and the stretching ratio is expressed in Figure 8, Figure 9 and Figure 10 on the temperature profile. Figure 9 shows the influence of volume concentration ϕ on the temperature profile h(η). An increment in volume fraction has shown a contraction in the thermal boundary layer for all examined nanofluids. In this case, Zn-water the nanofluid thermal boundary layer has shown high contraction compared to other explored nanofluids. Moreover, under the increasing influence of volume fraction, the temperature profile has decreased drastically.

It is perceived in Figure 10 that, as the temperature exponent A increases, the thermal boundary layer thickness also increases. The temperature exponent plays a key role in the expansion of the thermal boundary layer thickness. When the thermal exponent is raised −1<A≤0, the thermal boundary of free moving nanofluids upsurges sharply, but when the thermal exponent lands inbound 0<A≤1, it is clear from Figure 9 that the thermal boundary layer of all investigated nanofluids has decreased or contracted more rapidly. Figure 10 shows the relationship between the stretching ratio parameter and the temperature profile. It can be seen that the temperature profile h(η) of nanofluid increases as the stretching ratio parameter increases. Furthermore, the associated thermal boundary layer decreases with the incremental impression of the stretching ratio. This displays the impact of the bi-linear stretching ratio on the temperature profile in the presence of thermal exponent and low volume fraction. 

Table 1 and Table 2 demonstrate the complete agreement of the obtained outcomes of the heat transfer coefficient, termed the Nusselt number, with already published work. Table 1 shows the Nusselt number agreement when the thermal exponent and Prandtl number are varied while the stretching ratio remains constant. The outcomes were in complete agreement with those of Magyari and Keller and Liu et al. Table 2 also depicts a comparison of obtained outcomes. These results were accumulated under the varying stretching ratio. These outcomes were also verified with those of Magyari and Keller and Liu et al.

The thermophysical properties of the base fluid and solid nanoparticles are shown in Table 3. Table 4 provides the skin friction coefficients in the x− and y− direction $\left(f^{\prime \prime },\;g^{\prime \prime }\right)$, respectively, along with the heat transfer coefficient termed as the Nusselt number $\left(h^{\prime }\right)$. Table 4 indicates that when the temperature exponent A increases, the heat transfer coefficient increases for all nanofluid combinations. By rising the stretching ratio parameter γ, the heat transfer coefficient also increases. The skin coefficient reduced more rapidly in the y-direction compared to the skin force in the x-direction. When porosity parameter increases the velocity of the fluid increases because flow resistance decreases and. It is clearly, evident (Table 4) that Ag-water has a high-temperature transference rate as compared to other nanofluids.

## 6. Conclusions

In this research, the authors investigated the effect of various nanoparticle volumetric concentrations ϕ on 3D boundary layer flow using water as the base fluid across an exponentially stretching surface. By using a homogeneous flow model, the influences for Zn, Cu, and Ag are accounted for. The impacts of leading parameters, such as volumetric concentration, porosity H temperature exponent A and stretching ratio constraint γ on velocity profiles as well as temperature profiles are explored. The major discoveries of this research are the following:

(a) Boundary layer thickness has decreased more rapidly in the secondary velocity profile $g^{\prime }$ as compared to the primary velocity profile $f^{\prime }.$
(b) An increment in temperature exponent A results in the enhancement of heat transfer $\;Nu_{x}$ for all examined nanofluid and is highly noted for Ag-water nanofluid.
(c) Reduced skin friction coefficients $(C_{fx}$, $C_{fy})$ are obtained in *y*-direction compared to *x*-direction under different increasing study parameters.
(d) Skins frictions $(C_{fx}$, $C_{fy})$ and heat transfer $\;Nu_{x}$ coefficients have increased under the increasing influence of the stretching ratio.
(e) The porosity H decreases the skin friction and increases the fluid flow.
(f) Out of all examined nanofluids, namely, Ag-water, Zn-water, and Cu-water, reduced skin frictions and high heat transfer rates were observed for Ag-water under the different parametric influences.

## 7. Recommendations

In general, this study suggests more frequent exploitation of all examined nanofluids, especially Ag-water nanofluid. Reduced skin coefficients and enhanced heat transfer rates are obtained. Achieved outcomes demonstrate good agreement with already published data and with the advancement in nanofluid technology, it is recommended to explore more frequently the newly discovered phenomenon termed hybrid nanofluids.

## 8. Practical Significance

Choi and Eastman were the first who coined the term “nanofluids” in 1995. They introduced the heat enhancement of conventional fluids by distributing nanometer-sized particles into the host/base fluid. The thermal conductivity of the fluid was enhanced, and better heat exchange rates were obtained. After this innovation, nanofluid technology has a plethora of applications in the industrial and commercial sectors. Nanofluids have numerous applications in the heat transfer industry, such as plate heat exchangers, shell and tube heat exchangers, and compact and double pipe heat exchangers. Moreover, specifically under the obtained outcomes in this study, the examined nanofluid, Ag-water, has great potential to be used in flat plate solar collectors. Water can also be tested in natural convective flat plate solar collector systems under real solar effects.

## Figures and Tables

**Table 1:** Comparison of Nusselt number $\left(Nu_{x}\right)$ with [43,44] under increasing Prandtl number Pr and temperature exponent A with constant stretching ratio γ=0.

$h^{\prime }\left(0\right)\;or\;\;Nu_{x}$				
Pr	A	Present Results	Magyari and Keller [43]	Liu et al. [44]
1	−1.5	0.337462	0.3774	0.37741
	0	−0.557717	−0.5496	−0.54964
	1	−0.957645	−0.9547	−0.95478
	3	−1.560353	−1.5603	−1.56035
5	−1.5	1.35369	1.3533	1.35334
	0	−1.521283	−1.5212	1.52123
	1	−2.500211	−2.5001	−2.50013
	3	−3.88661	−3.8865	−3.88655
10	−1.5	2.20003	2.20000	2.20002
	0	−2.25751	−2.2574	−2.25742
	1	−3.66005	−3.6604	−3.66041
	3	−5.62821	−5.6353	−5.62819

**Table 2:** Comparison of Nusselt number $\left(Nu_{x}\right)$ with [44], when Prandtl number Pr=0.7 is and stretching ratio γ and temperature exponent A are increasing.

$h^{\prime }\left(\eta \right)\;or\;\;Nu_{x}$							
		A = −2		A = 0		A = 2	
γ	Pr	Liu et al. [44]	Present *Results*	*Liu et al.* [44]	Present Results	Liu et al. [44]	Present Results
0	0.7	0.6236	0.62371	−0.4258	−0.448803	−1.6416	−1.642714
	07	5.9409	5.94689	−1.8466	−1.84665	−5.8978	−5.90858
0.5	0.7	0.7637	0.764665	−0.5215	−0.535008	−2.0106	−2.02543
	07	7.2761	7.27621	−2.2616	−2.26168	−7.2233	−7.28729
1	0.7	0.8819	0.88241	−0.6022	−0.610684	−2.3216	−2.34461
	07	8.4017	8.41120	−2.6115	−2.61153	−8.3407	−8.35527

**Table 3:** Thermophysical properties of selected nanoparticles and pure base fluid (water) [4,7,9].

Properties	Water	Zn	Cu	Ag
$\rho \;\left(kg/m^{3}\right)$	997	7140	8933	10,490
$c_{p\;}\left(J/kgK\right)$	4179	389	385	235
k (w/mK)	0.613	120	400	429

**Table 4:** Numerical outcomes for skin frictions $(C_{fx}$, $C_{fy})$ and Nusselt number ${\;Nu}_{x}$ from the current analysis.

ϕ	A	γ	$\frac{f^{\prime \prime }\left(0\right)}{{\left(1-\phi \right)}^{5/2}}$	$\frac{g^{\prime \prime }\left(0\right)}{{\left(1-\phi \right)}^{5/2}}$	$\frac{-k_{nf}}{k_{f}}h^{\prime }\left(0\right)$
0.01	−0.5	0.5	−2.28079 (Zn)	−1.1404 (Zn)	2.03207 (Zn)
			−2.40341 (Cu)	−1.2017 (Cu)	2.15200 (Cu)
			−2.50509 (Ag)	−1.25255 (Ag)	2.24415 (Ag)
		1.5	−2.94071 (Zn)	−4.41106 (Zn)	2.62488 (Zn)
			−3.09962 (Cu)	−4.64943 (Cu)	2.77947 (Cu)
			−3.23131 (Ag)	−4.84697 (Ag)	2.89827 (Ag)
0.02	0.5	0.5	−3.10536 (Zn)	−1.55268 (Zn)	7.43356 (Zn)
			−3.34453 (Cu)	−1.67227 (Cu)	8.09202 (Cu)
			−3.5393 (Ag)	−1.76965 (Ag)	8.56881 (Ag)
		1.5	−4.00416 (Zn)	−6.00624 (Zn)	9.59943 (Zn)
			−4.31402 (Cu)	−6.47103 (Cu)	10.4489 (Cu)
			−4.56615 (Ag)	−6.84922 (Ag)	11.064 (Cu)

## Abbreviations

$U_{w},\;V_{w}$	Stretching rates of surface
3D	Three dimensional
$Cf_{x},\;Cf_{y}$	Skin friction alongside x-axis and y-axis
$C_{p}$	Specific heat capacity
$\rho C_{p}$	Volumetric heat capacity
k	Thermal conductivity
$\mu_{nf}$	Dynamic viscosity of nanofluid
L	Reference length
γ	Surface stretching ratio parameter
$\tau_{w}$	Wall shear stress
$Nu_{x}$	Nusselt number
Pr	Prandtl number
Re	Reynolds number
T	Temperature of fluid
MHD	Magneto hydrodynamic
$T_{w}$	Temperature at wall
$T_{\infty }$	The temperature outside the surface
u,v,w	Velocity constituents of x-, y-, and z-axis, respectively
$\alpha_{nf}$	Thermal diffusivity of nanofluid
**v_nf_**	Kinematics viscosity of nanofluid
η	Dimensionless variable
$\rho_{nf}$	Density of nanofluid
$q_{w}$	Wall temperature flux
ϕ	volume concentration of nanoparticles
f,s,nf	Subscripts for fluid, solid nanoparticles, and nanofluid, respectively
A	Temperature exponent
